# Supplementary material for: CEM500K, a large-scale heterogeneous unlabeled cellular electron microscopy image dataset for deep learning
Source: eLife. 2021 Apr 8;10:e65894. doi: 10.7554/eLife.65894 (PMC8032397; doi:10.7554/eLife.65894)
Supplement: Supplementary file 1. [file elife-65894-supp1.docx]

| **Benchmark** | **Publication Title** | **Year** | **Authors** | **Biological Context** | **Imaging Technique** | **Voxel Size (nm)** | **Dimensions** | **Training Set Pixels/Voxels** | **Test Set Pixels/Voxels** | **Segmentation Class(es)** |
| --- | --- | --- | --- | --- | --- | --- | --- | --- | --- | --- |
| All Mitochondria | N/A | N/A | N/A | Mouse Bladder, Mouse Brain & Human Platelets | SBF-SEM, FIB-SEM, ssSEM | 10x10x50, 5x5x5, 3x3x29, 30x30x30, 16x16x15 | 2D and 3D | 4.42E+08 | 3.71E+08 | Mitochondria |
| CREMI Synaptic Clefts | MICCAI Challenge on Circuit Reconstruction from Electron Microscopy Images | 2016 | J. Funke, S. Saalfeld, D. Bock, S. Turaga, E. Perlman | Drosophila Brain | ssTEM | 4x4x40 | 3D | 3.91E+08 | 1.95E+08 | Synaptic Clefts |
| Guay | Dense cellular segmentation for EM using 2D-3D neural network ensembles | 2020 | M. Guay, Z. Emam, A. Anderson, M. Aronova, and R. Leapman | Human Platelets | SBF-SEM | 10x10x50 | 3D | 3.20E+07 | 2.95E+07 | Mitochondria, Canalicular Channels, Alpha Granules, Dense Granules, Dense Granule Cores |
| Lucchi++ | Fast Mitochondria Segmentation For Connectomics | 2018 | V. Casser, K. Kang, H. Pfister and D. Haehn | Mouse Brain | FIB-SEM | 5x5x5 | 2D | 1.30E+08 | 1.30E+08 | Mitochondria |
| Kasthuri++ | Fast Mitochondria Segmentation For Connectomics | 2018 | V. Casser, K. Kang, H. Pfister and D. Haehn | Mouse Brain | ssSEM | 3x3x29 | 2D | 2.01E+08 | 1.55E+08 | Mitochondria |
| Perez | A workflow for the automatic segmentation of organelles in electron microscopy image stacks | 2014 | A. J. Perez et al. | Mouse Brain | SBF-SEM | 30x30x30 | 2D | 1.25E+07 | 4.00E+07 | Mitochondria, Lysosomes, Nuclei, Nucleoli |
| UroCell | Automatic segmentation of mitochondria and endolysosomes in volumetric electron microscopy data | 2020 | M. Žerovnik Mekuč et al. | Mouse Bladder | FIB-SEM | 16x16x15 | 3D | 6.71E+07 | 1.68E+07 | Mitochondria, Lysosomes |

**Supplementary File 1:** Characteristics of the benchmark datasets. Benchmark data can be accessed through the following links: [CREMI Synaptic Clefts](https://cremi.org/data/), [Guay](https://leapmanlab.github.io/dense-cell/), [Lucchi++](http://casser.io/connectomics/), [Kasthuri++](http://casser.io/connectomics/), [Perez](https://www.sci.utah.edu/download/chm/), [UroCell](https://github.com/MancaZerovnikMekuc/UroCell).

<https://cremi.org/data/>

<https://leapmanlab.github.io/dense-cell/>

<http://casser.io/connectomics/>

<http://casser.io/connectomics/>

<https://www.sci.utah.edu/download/chm/>

<https://github.com/MancaZerovnikMekuc/UroCell>
