# Supplementary material for: CEM500K, a large-scale heterogeneous unlabeled cellular electron microscopy image dataset for deep learning
Source: eLife. 2021 Apr 8;10:e65894. doi: 10.7554/eLife.65894 (PMC8032397; doi:10.7554/eLife.65894)
Supplement: Supplementary file 2. [file elife-65894-supp2.docx]

| **Benchmark** | **Training Iterations** | **CEM500K-moco pre-trained with benchmark data** | **CEM500K-moco pre-trained without benchmark data** |
| --- | --- | --- | --- |
| All Mitochondria | 10000 | **0.770** | 0.740 |
| CREMI Synaptic Clefts | 5000 | **0.254** | 0.238 |
| Guay | 1000 | **0.429** | 0.427 |
| Kasthuri++ | 10000 | **0.915** | 0.913 |
| Lucchi++ | 10000 | 0.895 | **0.903** |
| Perez | 2500 | 0.901 | **0.902** |
| Lysosomes | -- | **0.849** | 0.848 |
| Mitochondria | -- | **0.884** | 0.881 |
| Nuclei | -- | **0.988** | 0.985 |
| Nucleoli | -- | 0.885 | **0.894** |
| UroCell | 2500 | 0.734 | **0.758** |

**Supplementary File 2:** Comparison of segmentation IoU results for benchmark datasets from models pre-trained with MoCoV2 on versions of CEM500K that either included or excluded images from the benchmark datasets. * The best result for each benchmark is highlighted in bold and underlined.
