# Supplementary material for: CEM500K, a large-scale heterogeneous unlabeled cellular electron microscopy image dataset for deep learning
Source: eLife. 2021 Apr 8;10:e65894. doi: 10.7554/eLife.65894 (PMC8032397; doi:10.7554/eLife.65894)
Supplement: Supplementary file 3. [file elife-65894-supp3.docx]

| **Training Iterations** | **Cross-Entropy Weights** | **Gaussian Noise Aug.** | **Guay Train IoU 2D** | **Guay Validation IoU 2D** |
| --- | --- | --- | --- | --- |
| 5000 | **--** | **--** | 0.545 | 0.368 |
| 5000 | ✔︎ | **--** | 0.707 | 0.397 |
| 5000 | ✔︎ | ✔︎ | 0.609 | 0.384 |

**Supplementary File 3:** Validation results on the Guay dataset for different choices of training hyperparameters.
